# Relationship between coronary tortuosity and plateletcrit coronary tortuosity and plateletcrit

**DOI:** 10.5830/CVJA-2017-023

**Published:** 2017

**Authors:** Cerit Levent, Cerit Zeynep

**Affiliations:** Near East University Hospital, Nicosia, Cyprus; Near East University Hospital, Nicosia, Cyprus

**Keywords:** coronary angiography, plateletcrit, mean platelet volume

## Abstract

**Background:**

Coronary tortuosity (CorT) is a common angiographic finding and may be associated with myocardial ischaemia, even without coronary artery disease. Platelets play a crucial role in inflammatory and thrombotic processes and the physiopathology of cardiovascular disease. Larger platelets are more active enzymatically and have higher thrombotic ability compared to smaller platelets. Plateletcrit (PCT) provides complete information on total platelet mass. We aimed to evaluate the relationship between CorT and PCT in patients with chronic stable angina.

**Methods:**

The medical records of consecutive patients who underwent coronary angiography from January 2013 to January 2016 were retrospectively reviewed for CorT. CorT and clinical, echocardiographic, haematological and biochemical parameters were evaluated. Taking into consideration the inclusion criteria, 106 patients with CorT and 108 with normal coronary angiographies (control group) were included in the study. CorT was defined as three fixed bends during both systole and diastole, with each bend ≥ 45°.

**Results:**

The median PCT, mean platelet volume (MPV), platelet:large-cell ratio (P-LCR), neutrophil:lymphocyte ratio (NLR) and platelet:lymphocyte ratio (PLR) of the CorT group were significantly higher than those of the control group (0.26 ± 0.02 vs 0.2 ± 0.03%, p < 0.001; 10.6 ± 0.14 vs 9.6 ± 0.65 fl, p < 0.001; 29.3 ± 6.7 vs 23.4 ± 5.1, p < 0.001; 2.3 ± 1 vs 1.47 ± 0.48, p < 0.001; 1.28 ± 0.5 vs 0.82 ± 0.23, p < 0.001, respectively). The incidence of diabetes mellitus, hypertension and female gender were significantly higher in the CorT group (18.9 vs 1.9%, p < 0.001, 90.6 vs 50%, p < 0.001, 70.8 vs 44.4%, p < 0.001, respectively). Multivariate logistic regression analysis revealed age, hypertension, diabetes mellitus and plateletcrit were independently associated with CorT.

**Conclusion:**

CorT was associated with increased PCT, MPV, P-LCR, NLR and PLR, even in the absence of coronary artery disease. Age, hypertension, diabetes mellitus and plateletcrit were independently associated with CorT.

## Background

Coronary tortuosity (CorT) is a common coronary angiographic finding. CorT is defined as three fixed bends during both systole and diastole in at least one epicardial artery, with each bend showing a 45° change in vessel direction.^1^ The aetiology, clinical implications and long-term prognosis are not well clarified.

Clinical studies have demonstrated that CorT may be related to aging, hypertension, atherosclerosis and diabetes mellitus.^2^,^3^ CorT is associated with reversible myocardial perfusion defects and chronic stable angina.^4^,^5^ Zegers et al.6 demonstrated three cases of patients with CorT and hypothesised that CorT may lead to ischaemia. Patients with CorT may suffer from effortinduced chest pain and pain at rest.^6^ The relationship between CorT and coronary atherosclerosis is unclear.

Platelets play a key role in the genesis of thrombosis. Platelets, the amount of which in the blood is indicated with plateletcrit (PCT), are important for inflammation, thrombosis and cardiovascular pathophysiology. Increased mean platelet volume (MPV) has been found to be associated not only with coronary artery disease but also with carotid artery disease, deep-vein thrombosis and pulmonary embolism.^7^-^9^ Platelets with larger volumes are more active and vulnerable and therefore are a topic of interest in the development of atherosclerosis.10

PCT provides more comprehensive data about total platelet mass because it is equivalent to MPV and platelet count (PLT), where PCT = PLT × MPV/107. To the best of our knowledge, PCT and its association with CorT has not been previously reported. In this study, we investigated the relationship between all platelet markers and CorT.

## Methods

A retrospective evaluation was performed of consecutive patients undergoing coronary angiography due to stable angina pectoris. Stable angina was defined as discomfort in the chest, back, jaw, shoulder or arms, typically elicited by exertion or emotional stress, and relieved by rest or nitroglycerin. All patients enrolled in the study underwent coronary angiography as a result of chest pain and objective signs of ischaemia during treadmill exercises.

Routine laboratory and clinical parameters (hypertension, hypercholesterolaemia, diabetes mellitus and tobacco use) were obtained from the patient medical records. Study exclusion criteria included acute or chronic hepatic and renal failure, chronic obstructive pulmonary disease, peripheral and cerebral arterial disease, inflammatory diseases, congenital heart disease, restrictive cardiomyopathy, dilated cardiomyopathy, hypothyroidism, hyperthyroidism, malignancies, autoimmune diseases, acute or chronic infectious disease, coronary ectasia, severe coronary artery disease, coronary slow flow, mild-to-severe valvular disease, heart failure, anaemia and patients on anti-aggregation therapy.

All patients underwent coronary angiography according to the Judkins technique. Angiograms were reviewed by at least two blinded reviewing cardiologists. The left anterior descending coronary artery (LAD), left circumflex coronary artery (LCX) and right coronary artery (RCA) were observed in various angulations. CorT was evaluated on special angulations, the LAD was assessed in the right anterior oblique view with cranial angulations, and the LCX in the left anterior oblique with caudal angulations, while the RCA was assessed in the right anterior oblique view. CorT was identified by three or more bends (defined as ≥ 45° change in vessel direction) along the main trunk of at least one artery, present both in systole and diastole.^1^

Prior to coronary angiography, eight-hour postprandial venous blood was collected from all patients for routine laboratory testing. Haematological parameters, including haemoglobin, haematocrit and white blood cell count were analysed using an automated CBC device (Abbott Cell Dyn; Abbott Laboratories, Effingham, Illinois, USA). Biochemical parameters were measured using an Olympus AU 600 autoanalyser (Olympus Optical Co, Ltd, Schimatsu-Mishima, Japan). All study parameters were reviewed and approved by the local ethics committee.

## Statistical analysis

Statistical analysis was performed using the SPSS (version 20.0, SPSS Inc, Chicago, Illinois) software package. Continuous variables are expressed as mean ± standard deviation (mean ± SD) and categorical variables as percentages (%). The Kolmogorov– Smirnov test was used to evaluate the distribution of variables. The Student’s t-test was used to evaluate continuous variables showing normal distribution and the Mann–Whitney U-test was used to evaluate variables that did not show normal distribution. A p-value < 0.05 was considered statistically significant.

To identify predictors of CorT, the following variables were initially assessed in a univariate model: age, hypertension, female gender, diabetes, hyperlipidaemia, current smoking and haematological parameters. Significant variables in univariate analysis were then entered into a multivariate logistic regression analysis using backward stepwise selection.

## Results

The coronary tortuosity and normal coronary groups comprised 106 and 108 patients, respectively. The demographic characteristics of both groups are summarised in Table 1.

**Table 1:** General characteristics of the patients

*Patient characteristics*	Coronary tortuosity	
	*+ (n = 106)*	*(n = 108)*	*p-value*
Age, years	61.8 ± 6.1 (61)	52.9 ± 8.1 (51)	< 0.001
Female gender, n (%)	75 (70.8)	48 (44.4)	< 0.001
Hypertension, n (%)	96 (90.6)	54 (50)	< 0.001
Diabetes mellitus, n (%)	20 (18.9)	2 (1.9)	< 0.001
Current smoking, n (%)	30 (28.3)	22 (20.4)	0.176

There was no significant difference between the groups regarding current smoking (28.3 vs 20.4% p = 0.176) (Table 1). There was no significant difference between the groups regarding total cholesterol, high-density lipoprotein cholesterol and triglyceride levels, haemoglobin, white blood cell and neutrophil counts, C-reactive protein, urea and creatinine levels, and ejection fraction [209.9 ± 30.5 vs 206.3 ± 31.8 mg/dl (5.44 ± 0.79 vs 5.34 ± 0.82 mmol/l), p = 0.478; 48 ± 13.6 vs 43.3 ± 5.6 mg/ dl (1.24 ± 0.35 vs 1.12 ± 0.12 mmol.l), p = 0.075; 152.4 ± 27.4 vs 163.1 ± 48.5 mg/dl (1.72 ± 0.31 vs 1.84 ± 0.55 mmol/l), p = 0.517; 13.3 ± 1.5 vs 14.4 ± 1.2 g/dl, p = 0.527; 6.96 ± 2.12 vs 7.0 ± 1.3 103/µl, p = 0.683; 4.35 ± 1.6 vs 3.97 ± 0.99 103/µl, p = 0.408; 0.48 ± 0,14 vs 0.44 ± 0.18 mg/dl, p = 0.267; 35.0 ± 10.5 vs 38.7 ± 5.8 mg/ dl, p = 0.428; 0.79 ± 0.18 vs 0.83 ± 0.12 mg/dl; p = 0.367; 60.4 ± 2.5 vs 61.6 ± 3.2%, p = 0.751, respectively] (Table 2).

**Table 2:** Laboratory and echocardiographic parameters

*Laboratory and echocardiographic variables*	*Coronary tortuosity*	
	*+ (n = 106)*	*(n = 108)*	*p-value*
Haemoglobin (g/dl)	13.3 ± 1.5 (13.9)	14.4 ± 1.2 (14.1)	0.527
Platelets (103/µl)	236.5 ± 57.1 (229.0)	218.5 ± 32.1 (219.0)	0.009
White blood cells (103/µl)	6.96 ± 2.12 (6.84)	7.0 ± 1.3 (6.9)	0.683
Mean platelet volume (fl)	10.6 ± 1.4 (10.5)	9.5 ± 0.65 (9.5)	< 0.001
Neutrophils (103/µl)	4.35 ± 1.6 (4.1)	3.97 ± 0.99 (4.39)	0.408
Lymphocytes (103/µl)	1.99 ± 1 (1.96)	2.77 ± 0.58 (2.65)	< 0.001
Neutrophil:lymphocyte Ratio	2.3 ± 1 (2.06)	1.47 ± 0.48 (1.45)	< 0.001
Platelet:large-cell ratio	29.3 ± 6.7 (29)	23.4 ± 5.1 (23.1)	< 0.001
Platelet:lymphocyte ratio	1.28 ± 0.5 (1.1)	0.82 ± 0.23 (0.8)	< 0.001
Plateletcrit (%)	0.26 ± 0.16 (0.24)	0.21 ± 0.03 (0.20)	< 0.001
C-reactive protein (mg/dl)	0.48 ± 0.14 (0.45)	0.44 ± 0.18 (0.41)	0.267
Total cholesterol (mg/dl)	209.9 ± 30.5 (210)	206.3 ± 31.8 (204)	0.478
[mmol/l]	[5.44 ± 0.79 (5.44)]	[5.34 ± 0.82 (5.28)]	
High-density lipoprotein cholesterol (mg/dl)	48 ± 13.6 (45)	43.3 ± 5.9 (43)	0.075
[mmol/l]	[1.24 ± 0.35 (1.17)]	[1.12 ± 0.15 (1.11)]
Low-density lipoprotein cholesterol (mg/dl)	139.9 ± 24.7 (148)	132.7 ± 27.4 (134)	0.02
[mmol/l]	[3.62 ± 0.64 (3.83)]	[3.44 ± 0.71 (3.47)]
Triglycerides (mg/dl)	152.4 ± 27.4 (134)	163.1 ± 48.5 (154.5)	0.517
[mmol/l]	[1.72 ± 0.31 (1.51)]	[1.84 ± 0.55 (1.75)]
Urea (mg/dl)	35.0 ± 10.5 (36)	38.7 ± 5.8 (38)	0.428
Creatinine (mg/dl)	0.79 ± 0.18 (0.76)	0.83 ± 0.12 (0.80)	0.367
Ejection fraction (%)	60.4 ± 2.5 (61.1)	61.6 ± 3.2 (62.4)	0.751

However, there was a significant difference between the groups regarding hypertension, with more hypertensive patients in the CorT group (90.6 vs 50%, p < 0.001) (Table 1). There were also significant differences between the groups regarding age, female gender, diabetes mellitus and low-density lipoprotein cholesterol levels [61.8 ± 8.7 vs 52.9 ± 8.1 years, p < 0.001; 70.8 vs 44.4%, p < 0.001; 18.9 vs 1.9%, p < 0.001; 139.9 ± 24.7 vs 132.7 ± 27.4 mg/dl (3.62 ± 0.64 vs 3.44 ± 0.71 mmol/l), p = 0.02, respectively].

The median PCT, MPV, P-LCR, NLR and PLR values of the CorT group were significantly higher than those of the control group (0.26 ± 0.02 vs 0.2 ± 0.03%, p < 0.001; 10.6 ± 0.14 vs 9.6 ± 0.65 fl, p < 0.001; 29.3 ± 6.7 vs 23.4 ± 5.1, p < 0.001; 2.3 ± 1 vs 1.47 ± 0.48, p < 0.001; 1.28 ± 0.5 vs 0.82 ± 0.23, p < 0.001, respectively) (Table 2).

The results of univariate analyses are presented in Table 3. On univariate analysis, age, diabetes mellitus, hypertension, female gender, PCT and NLR were associated with CorT (Table 3). On multivariate analysis age, hypertension, diabetes mellitus and PCR were independent predictors for CorT (OR 1.826; 95% CI: 1.354–2.167; p < 0.001, OR 2.158; 95% CI: 1.462–2.937; p < 0.001, OR 1.583; 95% CI: 1.362–2.835; p < 0.001, OR 1.634; 95% CI: 1.345–2.724; p < 0.001, respectively) (Table 4).

**Table 3:** Univariate analysis of predictors for coronary tortuosity

*Predictor variables*	*OR (95% CI)*	*p-value*
Age, years	3.275 (1.943–5.627)	< 0.001
Diabetes mellitus, n (%)	2.539 (1.675–3.592)	< 0.001
Hypertension, n (%)	2.856 (1.345–3.863)	< 0.001
Female gender, n (%)	2.348 (1.857–4.362)	< 0.001
Plateletcrit	2.896 (1.964–4.857)	< 0.001
Neutrophil:lymphocyte ratio	1.854 (1.376–2.827)	0.001

**Table 4:** Multivariate analysis of predictors for coronary tortuosity

Predictor variables	OR (95% CI)	p-value
Age, years	1.826 (1.354–2.167)	< 0.001
Hypertension, n (%)	2.158 (1.462–2.937)	< 0.001
Diabetes mellitus, n (%)	1.583 (1.362–2.835)	< 0.001
Plateletcrit	1.634 (1.345–2.724)	< 0.001

## Discussion

In this study, we investigated the association between CorT and PCT. Our results reveal that PCT, MPV, P-LCR, NLR and PLR of patients with CorT were higher than those of the control group consisting of patients with a normal coronary artery. Age, hypertension, diabetes mellitus and PCT were independently associated with CorT.

CorT is a common coronary angiographic finding. In the study by Li, et al., the prevalence of CorT was 39.1% in patients with stable angina pectoris.^3^ To date the aetiology of CorT is unclear. There are several possible mechanisms implicated in the development of CorT. Some authors claim that degeneration of the elastin layer of the vessel may be the cause of coronary tortuosity.^11^ CorT may be associated with age, hypertension and atherosclerosis.12-14 In our study, CorT was independently associated with hypertension, diabetes mellitus, age and PCT.

Li et al.^3^ found that CorT was positively correlated with essential hypertension. They hypothesised that the arteries may become tortuous due to reduced axial strain and hypertensive pressure in an elastic cylindrical arterial model. Therefore CorT may be one of the forms of artery remodelling induced by hypertension due to increased coronary pressure and blood flow. 

This is consistent with the findings of our study. We found a highly significant difference between the CorT and non-CorT groups regarding the presence of hypertension. However, some authors suggest that CorT is a common finding seen with aging and hypertension due to elongation and dilatation of the arteries associated with left ventricular hypertrophy.^13^,^15^

CorT may be related to typical anginal pain with angiographic evidence of objective ischaemia without significant coronary lesions. This could be due to compression of the vessel during heart contraction.^14^ CorT has a minor influence on coronary blood supply at rest, whereas during exercise, patients with CorT may lack the ability to adjust distal resistance sufficiently to compensate for the extra resistances generated by tortuosity, and this may further lead to an ineffective regulation of the blood supply.^16^

Li et al.^17^ found that CorT can result in decreased coronary blood pressure depending on the severity of tortuosity, and therefore severe CorT may cause myocardial ischaemia. In another study, Li et al.^5^ found that CorT was associated with reversible myocardial perfusion defects in patients with chronic stable angina and normal coronary angiograms.

CorT is associated with coronary atherosclerotic changes regardless of the presence of actual coronary stenosis. CorT may induce subclinical atherosclerosis in the absence of significant obstructive lesions.^18^ Severe tortuosity in the coronary arteries simplifies atherosclerosis.^19^ Therefore atherosclerosis is more common in patients with coronary artery tortuosity, as greater curvature has more areas of low-shear wall stress. Shear stress is an essential causal factor in the development of atherosclerosis^20^ and in vulnerable plaque rupture.^21^ Davutoglu et al.^22^ found that CorT was strongly associated with subclinical atherosclerosis indicated by carotid intima–media thickness and retinal artery tortuosity.

On the other hand, some studies found that CorT was negatively correlated with significant coronary artery disease detected by coronary angiography.^3^,^23^ Esfahani et al.^24^ showed that the mean Gensini index of the tortuous group was significantly lower than that of the non-tortuous group.

Platelet activation plays a significant role in the initiation and progression of atherosclerosis.^25^,^26^ Platelets release many mediators such as thromboxanes, and interleukin (IL)-1, IL-3 and IL-6 that may lead to increased inflammation.^27^

PCT is part of the routine CBC haematology and provides more comprehensive data about total platelet mass because the PCT is the product of the platelet count and the MPV.^28^ Ekici et al.^29^ reported a strong association between MPV and angiographic severity of coronary artery disease. Several studies have shown that there was a strong relationship between PCT and saphenous vein disease and slow coronary flow.^30^,^31^ The association between haematological parameters and adverse cardiovascular outcomes has been shown in previous studies.^32^-^35^

 In this study, we found that PCT, MPV, NLR and PLR of the CorT group were significantly higher than those of the control group. Hypertension, diabetes mellitus, age and PCT were independently associated with CorT.

Our study has some limitations. First was the small sample size. Second, coronary angiography, which we used, only shows the arterial lumen, whereas cardiac computed tomography (CT) angiography and intravascular ultrasound (IVUS) allow visualisation of the lumen as well as the vascular wall. Cardiac CT and IVUS allow detection and characterisation of coronary atherosclerotic plaques. Accordingly, cardiac CT helps in the evaluation of atherosclerotic plaques that are undetected by conventional coronary angiography.

## Conclusions

This is the first study to evaluate the relationship between CorT and PCT. Hypertension, diabetes mellitus, age and PCT were independently associated with CorT. We concluded that CorT is associated with increased pro-inflammatory processes related to coronary artery disease. Long-term follow up of PCT levels in patients with CorT with regard to the development of coronary artery disease may be useful.

## References

[1] Turgut O, Yilmaz A, Yalta K, Yilmaz BM, Ozyol A, Kendirlioglu O (2007). Tortuosity of coronary arteries: an indicator for impaired left ventricular relaxation?. Int J Cardiovasc Imaging.

[2] Han HC (2012). Twisted blood vessels: symptoms, etiology and biomechanical mechanisms. J Vasc Res.

[3] Li Y, Shen C, Ji Y, Feng Y, Ma G, Liu N (2011). Clinical implication of coronary tortuosity in patients with coronary artery disease. PLoS One.

[4] Gaibazzi N, Rigo F, Reverberi C (2011). Severe coronary tortuosity or myocardial bridging in patients with chest pain, normal coronary arteries, and reversible myocardial perfusion defects. Am J Cardiol.

[5] Li Y, Liu NF, Gu ZZ, Chen Y, Lu J, Feng Y (2012). Coronary tortuosity is associated with reversible myocardial perfusion defects in patients without coronary artery disease. Chin Med J (Engl).

[6] Zegers ES, Meursing BT, Zegers EB, Oude Ophuis AJ (2007). Coronary tortuosity: a long and winding road. Neth Heart J.

[7] Valkila EH, Salenius JP, Koivula TA (1994). Platelet indices in patients with occlusive carotid artery disease. Angiology.

[8] Kostrubiec M, Labyk A, Pedowska-Wloszek J, Hrynkiewicz-Szymańska A, Pacho S, Jankowski K (2010). Mean platelet volume predicts early death in acute pulmonary embolism. Heart.

[9] Cil H, Yavuz C, Islamoglu Y, Tekbas EÖ, Demirtas S, Atilgan ZA (2012). Platelet count and mean platelet volume in patients with in-hospital deep venous thrombosis. Clin Appl Thromb Hemost.

[10] Yazici HU, Poyraz F, Sen N, Tavil Y, Turfan M, Tulmaç M (2011). Relationship between mean platelet volume and left ventricular systolic function in patients with metabolic syndrome and ST-elevation myocardial infarction. Clin Invest Med.

[11] Jakob M, Spasojevic D, Krogmann ON, Wiher v, Hug R, Hess OM (1996). Tortuosity of coronary arteries in chronic pressure and volume overload. Cathet Cardiovasc Diagn.

[12] Pancera P, Ribul M, Presciuttini B, Lechi A (2000). Prevalence of carotid artery kinking in 590 consecutive subjects evaluated by Echocolordoppler. Is there a correlation with arterial hypertension?. J Intern Med.

[13] Del  Corso L, Moruzzo D, Conte B, Agelli M, Romanelli AM, Pastine F (1998). Tortuosity, kinking, and coiling of the carotid artery: expression of atherosclerosis or aging?. Angiology.

[14] Satish G, Nampoothiri S, Kappanayil M (2008). Images in cardiovascular medicine. Arterial tortuosity syndrome: phenotypic features and cardiovascular manifestations. Circulation.

[15] Hutchins GM, Bulkley BH, Miner MM, Boitnott JK (1977). Correlation of age and heart weight with tortuosity and caliber of normal human coronary arteries. Am Heart J.

[16] Xie X, Wang Y, Zhu H, Zhou H, Zhou J (2013). Impact of coronary tortuosity on coronary blood supply: a patient-specific study. PLoS One.

[17] Li Y, Shi Z, Cai Y, Feng Y, Ma G, Shen C (2012). Impact of coronary tortuosity on coronary pressure: numerical simulation study. PLoS One.

[18] El Tahlawi M, Sakrana A, Elmurr A, Gouda M, Tharwat M (2016). The relation between coronary tortuosity and calcium score in patients with chronic stable angina and normal coronaries by CT angiography. Atherosclerosis.

[19] Cunningham KS (2005). The role of shear stress in the pathogenesis of atherosclerosis. Lab Invest.

[20] Wentzel JJ, Corti R, Fayad ZA, Wisdom P, Macaluso F, Winkelman MO (2005). Does shear stress modulate both plaque progression and regression in the thoracic aorta? Human study using serial magnetic resonance imaging. J Am Coll Cardiol.

[21] Santos-Gallego C.G, Picatoste B, Badim J.J (2014). Pathophysiology of acute coronary syndrome. Atheroscler Rep.

[22] Davutoglu V, Dogan A, Okumus S, Demir T, Tatar G, Gurler (2013). Coronary artery tortuosity: comparison with retinal arteries and carotid intima–media thickness. Kardiol Pol.

[23] Groves SS, Jain AC, Warden BE, Gharib W (2009). Beto RJ 2nd. Severe coronary tortuosity and the relationship to significant coronary artery disease. WV Med J.

[24] Esfahani M, Farzamnia H, Nezarat (2012). Chronic stable angina patients with tortuous coronary arteries: clinical symptoms and risk factors. ARYA Atheroscler J.

[25] Furman MI, Benoit SE, Barnard MR, Valeri CR, Borbone ML, Becker RC (1998). Increased platelet reactivity and circulating monocyte-platelet aggregates in patients with stable coronary artery disease. J Am Coll Cardiol.

[26] Ault KA, Cannon CP, Mitchell J, McCahan J, Novotny RP, Tracy WF (1999). Platelet activation in patients after an acute coronary syndrome: results from the TIMI-12 trial: thrombolysis in myocardial infarction. J Am Coll Cardiol.

[27] Klinger MH, Jelkmann W (2002). Role of blood platelets in infection and inflammation. J Interferon Cytokine Res.

[28] Ergelen M, Uyarel H (2014). Plateletcrit: a novel prognostic marker for acute coronary syndrome. Int J Cardiol.

[29] Ekici B, Erkan AF, Alhan A, Sayın I, Aylı M, Töre HF (2013). Is mean platelet volume associated with the angiographic severity of coronary artery disease?. Kardiol Pol.

[30] Akpinar I, Sayin MR, Gursoy YC, Aktop Z, Karabag T, Kucuk E (2014). Plateletcrit and red cell distribution width are independent predictors of the slow coronary flow phenomeno. J Cardiol.

[31] Akpinar I, Sayin MR, Gursoy YC, Karabag T, Kucuk E, Buyukuysal MC (2014). Plateletcrit: a platelet marker associated with saphenous vein graft diseas. Herz.

[32] Aydınlı B, Demir A, Güçlü ÇY, Bölükbaşı D, Ünal EU, Koçulu R (2016). Hematological predictors and clinical outcomes in cardiac surgery. J Anesth.

[33] Durukan AB, Gurbuz HA, Unal EU, Tavlasoglu M, Durukan E, Salman N (2014). Role of neutrophil/lymphocyte ratio in assessing the risk of postoperative atrial fibrillation. J Cardiovasc Surg (Torino).

[34] Unal EU, Ozen A, Kocabeyoglu S, Durukan AB, Tak S, Songur M (2013). Mean platelet volume may predict early clinical outcome after coronary artery bypass grafting. J Cardiothorac Surg.

[35] Unal EU, Durukan AB, Ozen A, Kubat E, Kocabeyoğlu SS, Yurdakok O (2013). Neutrophil/lymphocyte ratio as a mortality predictor following coronary artery bypass surgery. Turk Gogus Kalp Dama.

